# The use of mechanical insufflation-exsufflation in invasively ventilated critically ill adults: a scoping review protocol

**DOI:** 10.1186/s13643-020-01547-8

**Published:** 2020-12-08

**Authors:** Ema Swingwood, Willemke Stilma, Lyvonne Tume, Fiona Cramp, Frederique Paulus, Marcus Schultz, Wilma Scholte op Reimer, Louise Rose

**Affiliations:** 1 Faculty of Health and Social Care, University of the West of England, Bristol, UK; 2 Adult Therapy Services A804, Bristol Royal Infirmary, University Hospitals Bristol and Weston NHS Foundation Trust, Upper Maudlin Street, Bristol, BS2 8HW UK; 3 Faculty of Health, Center of Expertise Urban Vitality, Amsterdam University of Applied Sciences, Amsterdam, the Netherlands; 4 Department of Intensive Care Medicine, Amsterdam University Medical Centers, location ‘AMC’, Meibergdreef 9, 1105 AZ Amsterdam, the Netherlands; 5 School of Health and Society, University of Salford, Manchester, UK; 6 Alder Hey Children’s NHS Foundation Trust, Liverpool, UK; 7 Faculty of Health and Applied Sciences, University of the West of England, Bristol, UK; 8 Laboratory of Experimental Intensive Care and Anesthesiology (L·E·I·C·A), Amsterdam University Medical Centers, location ‘AMC’, Amsterdam, the Netherlands; 9 Mahidol–Oxford Tropical Medicine Research Unit (MORU), Mahidol University, Bangkok, Thailand; 10 Nuffield Department of Medicine, University of Oxford, Oxford, UK; 11 Department of Cardiology, Amsterdam University Medical Centers, location ‘AMC’, Amsterdam, the Netherlands; 12 Florence Nightingale Faculty of Nursing, Midwifery and Palliative Care, King’s College London, London, UK; 13 Lane Fox Respiratory Unit, Guy’s and Thomas’ Foundation NHS Hospital Trust, London, UK

**Keywords:** Mechanical insufflation-exsufflation, Intensive care, Critically ill adult, Invasively ventilated adult

## Abstract

**Background:**

Critically ill patients receiving invasive ventilation are at risk of sputum retention. Mechanical insufflation-exsufflation (MI-E) is a technique used to mobilise sputum and optimise airway clearance. Recently, interest has increased in the use of mechanical insufflation-exsufflation for invasively ventilated critically ill adults, but evidence for the feasibility, safety and efficacy of this treatment is sparse.

The aim of this scoping review is to map current and emerging evidence on the feasibility, safety and efficacy of MI-E for invasively ventilated adult patients with the aim of highlighting knowledge gaps and identifying areas for future research. Specific research questions aim to identify information informing indications and contraindications to the use of MI-E in the invasively ventilated adult, MI-E settings used, outcome measures reported within studies, adverse effects reported and perceived barriers and facilitators to using MI-E reported.

**Methods:**

We will search electronic databases MEDLINE, EMBASE, CINAHL using the OVID platform, PROSPERO, The Cochrane Library, ISI Web of Science and the International Clinical Trials Registry Platform. Two authors will independently screen citations, extract data and evaluate risk of bias using the Mixed Methods Appraisal Tool. Studies included will present original data and describe MI-E in invasively ventilated adult patients from 1990 onwards. Our exclusion criteria are studies in a paediatric population, editorial pieces or letters and animal or bench studies. Search results will be presented in a PRISMA study flow diagram. Descriptive statistics will be used to summarise quantitative data. For qualitative data relating to barriers and facilitators, we will use content analysis and the Theoretical Domains Framework (TDF) as a conceptual framework. Additional tables and relevant figures will present data addressing our research questions.

**Discussion:**

Our findings will enable us to map current and emerging evidence on the feasibility, safety and efficacy of MI-E for invasively ventilated critically ill adult patients. These data will provide description of how the technique is currently used, support healthcare professionals in their clinical decision making and highlight areas for future research in this important clinical area.

**Systematic review registration:**

Open Science Framework submitted on 9 July 2020. https://osf.io/mpksq/.

## Background

Critically ill patients under invasive ventilation are at risk for sputum retention [1]. The relatively dry gases used during invasive ventilation cause airway mucosa to produce more mucus volume, potentially of increased viscosity [1]. Cough is an important defence mechanism to clear mucus from the upper and lower airways [1]. The presence of an endotracheal tube impairs the ability to cough as the vocal cords and glottis cannot be closed. This prevents the generation of high intrathoracic pressure and subsequent enhancement of cough velocity [2, 3]. Furthermore, critically ill patients frequently have an impaired or no cough reflex due to depressed levels of consciousness, sedation, muscle weakness or muscle paralysis. Sputum retention, resulting from an inability to cough effectively, is one cause of extubation failure which in turn is associated with increased mortality [4].

There are a number of techniques to mobilise sputum and optimise airway clearance for invasively ventilated patients. Endotracheal suctioning is the most common intervention used to remove retained airway secretions from within the endotracheal tube, trachea and upper airways [5]. Endotracheal suctioning though is not effective for clearing secretions from the lower airways [6].

Mechanical insufflation-exsufflation (MI-E) aids sputum clearance from upper and lower airways. This technique augments inspiratory and expiratory flows to improve sputum mobilisation, through the application of rapidly alternating positive and negative pressure, which approximates a normal cough [7].

MI-E was originally developed to prevent respiratory complications associated with sputum retention for patients with neuromuscular disease [8, 9]. Recently interest has increased in the use of MI-E for invasively ventilated critically ill adults in the intensive care unit (ICU) [10]. To date, evidence suggests limited and variable adoption of MI-E in this patient group. Our group has conducted practice surveys of cough augmentation techniques in ICUs in Canada [11, 12], the United Kingdom (UK) [13] and the Netherlands [14]. Results from all surveys illustrated that MI-E was predominantly used for sputum management in non-intubated patients to prevent intubation or reintubation [11–13]. Across all three countries, MI-E was not commonly used in invasively ventilated patients. Both Canadian and UK surveys cited lack of clinician expertise and knowledge as perceived barriers to MI-E use in intubated patients.

Evidence for the feasibility, safety and efficacy of MI-E in invasively ventilated critically ill adults is sparse [15]. To date, little is known about which patients would benefit most and in which stage of mechanical ventilation, i.e. before or during weaning or following extubation to prevent reintubation; the most appropriate technique or MI-E set up regarding pressure, flow and timing of insufflation and exsufflation; incidence of adverse events; reported outcomes; and the barriers and facilitators for using MI-E for invasively ventilated adults in an ICU setting.

The primary aim of this scoping review is to map current and emerging evidence on how to use MI-E for invasively ventilated adult patients with the aim of highlighting knowledge gaps and identifying areas for future research.

## Methods

### Study design

Scoping review following the methods outlined by Arksey and O’Malley and advanced by other authors [16–18].

### Study questions

We will address the following study questions:

1. What primary clinical ICU diagnoses and/or reasons for mechanical ventilation are an indication to use/not use MI-E during invasive ventilation?
2. What are the clinical indications (i.e. sputum removal) and contraindications for commencing MI-E in invasively ventilated critically ill adults?
3. What MI-E settings are used for invasively ventilated critically ill adults? (i.e. interface type, flow, pressure and time settings)
4. What outcomes are reported in studies of MI-E for invasively ventilated critically ill adults and how are these outcomes measured?
5. What adverse events attributed to MI-E use are reported in the evidence base, and how are these defined/described?
6. What perceived barriers and facilitators to using MI-E for invasively ventilated critically ill adults are described in the evidence base, and how are these defined?

### Identifying relevant studies

The search strategy will be developed in consultation with a medical information specialist and applied to the following bibliographic electronic databases: MEDLINE, EMBASE and CINAHL using the OVID platform. We will search PROSPERO and The Cochrane Library for relevant reviews, ISI Web of Science for conference abstracts and the International Clinical Trials Registry Platform (apps.who.int/trialsearch) for unpublished and ongoing trials. We will screen reference lists of included articles for additional studies meeting our inclusion criteria listed below.

A modified version of the published search strategy of the Cochrane systematic review of cough augmentation techniques will be used [15]. Modification was made to solely focus on MI-E in an adult population. Additionally, we will not exclude studies based on study design. The search strategy is provided in Additional file 1. We will not restrict article selection based on language. Inclusion and exclusion criteria are shown in Table 1.

**Table 1:** Inclusion and exclusion criteria for studies

Inclusion	Exclusion
Mechanically ventilated adults via tracheostomy or endotracheal tube in a relevant clinical location (intensive care, weaning centres, respiratory high care/dependency areas)	Children (< 18 years)
Describes use of MI-E	Editorial pieces Letters to the Editor
Any study design (include randomised controlled trials (RCT), quasi and non-randomised clinical trials, before and after studies, interrupted time series cohort studies, qualitative designs, mixed methods, cross-sectional design, case reports/series, and research letters which present original data)	Bench and animal studies
Published from 1990 onwards	

### Selection of studies

Two review authors (ES and WS) will independently screen titles and abstracts identified by our search methods. Full texts of studies considered by either author as potentially eligible will be obtained and reviewed to confirm selection against the inclusion/exclusion criteria. Any disagreements throughout the review process will be resolved by discussion or referred to a third reviewer for arbitration (LR/FP). Endnote x9 will be used to select articles independently.

### Data charting process

The research team has developed the data charting form [17, 19] to collect information pertinent to our research questions. The data charting tool will be piloted by two authors (ES and WS) on five articles, with further refinement following discussion as required. Data will include article study demographics (author, year of publication, study location and population); study design and aim; primary clinical ICU diagnoses or reasons for mechanical ventilation of patients that use/do not use MI-E during invasive ventilation (RQ1); clinical indications and contraindications for using MI-E (RQ2); technical or practical application of MI-E (RQ3); study outcomes and measures (RQ4); adverse events/side effects (RQ5); and perceived barriers and facilitators to use of MI-E for invasively ventilated patients (RQ6).

Two reviewers (ES and WS) will independently chart these data using the data charting form. Data charting will be managed by two reviewers (WS and ES).

One reviewer will be responsible for contacting key author when clarification or additional data are needed. Contact efforts will be limited to a maximum of 3 emails.

### Analysis of data

Three steps will be used to collate results [17]. Descriptive statistics will be used to summarise quantitative data. We will present counts and proportions of studies reporting each outcome that have been used by researchers. For qualitative data relating to barriers and facilitators, we will use content analysis and the Theoretical Domains Framework (TDF) as a conceptual framework [20, 21]. Finally, we will apply meaning to the results through the generation of recommendations for practice and future research based on our analyses.

### Assessment of methodological quality of individual studies

Although the assessment of risk of bias is not essential for scoping reviews [18], we will use the Mixed Methods Appraisal Tool (MMAT) [22] to give an overview of the validity of current evidence. Previous studies have shown the MMAT to be an easy to use tool with moderate to perfect inter-rater reliability [22]. Two review authors (ES/WS) will independently complete quality assessment. We will not exclude studies from the review due to determined quality. Quality assessment instead will be used to facilitate description of rigour of included studies.

### Presentation of findings

We will present our search results in a PRISMA study flow diagram [18] illustrating the total number of articles generated from the search strategy and following application of the inclusion/exclusion criteria, the number subsequently excluded and ultimately used for review.

A summary table will illustrate study characteristics from included articles, including population, study country, study design and methods. Additional tables and relevant figures will present data addressing our research questions. Where qualitative data is attained, tables will be produced to highlight key thematic content within each TDF domain.

### Amendments

The protocol will be closely followed throughout with regular progress reports as a whole study team. If any amendments are made to the published study protocol, these will be reported in the final publication.

### Dissemination of findings

We plan to disseminate results from this review in a peer-reviewed journal.

## Discussion

There is growing interest in the role of MI-E for invasively ventilated critically ill adults but to date adoption and application of this technique is variable [11–13]. The primary aim of this scoping review is to map emerging and current evidence, on MI-E in an ICU setting, thus adding to previous Cochrane Review findings [15]. Our protocol also aims to apply the TDF framework to explore the perceived barriers and facilitators for MI-E use [20, 21]. Barriers and facilitators will be considered for the feasibility of this technique.

The results of this review will highlight gaps in the current evidence base to inform future research and will contribute to the clinical decision making processes of healthcare professionals who work with MI-E or are considering use of the technique within their ICU.

### Strength and limitations

The protocol for this scoping review is transparent and in line with the PRISMA scoping review checklist [18] and the recent scoping review checklist [23]. Strengths include rigorous and systematic search, inclusion of studies in all languages, independent selection of studies and quality assessment using the MMAT [22].

A potential limitation is that we are focusing on a very specific patient group with an age restriction. This may restrict the amount of articles to be included.

## Conclusion

This scoping review will provide a timely overview of emerging evidence of MI-E in invasively ventilated critically ill adults. We hope findings will facilitate clinician understanding the potential application of this technique for invasively ventilated critically ill adults and will direct future research.

## Supplementary Information

**Additional file 1:.** Search strategy

## Data Availability

The datasets used and/or analysed during the current study are available from the corresponding author on reasonable request.

## Abbreviations

MI-E: Mechanical insufflation-exsufflation
ICU: Intensive care unit
RQ: Research question
TDF: Theoretical Domains Framework
MMAT: Mixed Methods Appraisal Tool
PRISMA: Preferred Reporting Items for Systematic Reviews and Meta-Analyses
