# Supplementary material for: The use of mechanical insufflation-exsufflation in invasively ventilated critically ill adults: a scoping review protocol
Source: Syst Rev. 2020 Dec 8;9:287. doi: 10.1186/s13643-020-01547-8 (PMC7724723; doi:10.1186/s13643-020-01547-8)
Supplement: Supplementary file 1 — Additional file 1:. Search strategy [file 13643_2020_1547_MOESM1_ESM.docx]

*Additional file 1*

*Search strategy for the use of mechanical insufflation-exsufflation in invasively ventilated critically ill patients: a scoping review*

UPDATE: 24-2-2020 t/m 15-6-2020

**15-6-2020:**

| Databases: |  |  |  |
| --- | --- | --- | --- |
| Medline. Embase, Cinahl, Central, Web of Science | Before deduplication | After deduplication | After deduplication original document |
| Total | 128 | 112 | 76 |

Searches Before deduplication:

MEDLINE (OVID):

Database(s): **Ovid MEDLINE(R) and Epub Ahead of Print, In-Process & Other Non-Indexed Citations and Daily**1946 to June 12, 2020
Search Strategy:

| **#** | **Searches** | **Results** |
| --- | --- | --- |
| 1 | (cough* adj2 assist*).ti,ab,kw. | 261 |
| 2 | (CoughAssist* or Pegaso* or Cofflator* or Cof-flator* or cough machine*).ti,ab,kw. | 64 |
| 3 | (cough* adj2 augment*).ti,ab,kw. | 79 |
| 4 | Cough/rh [Rehabilitation] | 19 |
| 5 | (in-exsufflator* or in-exsufflation*).ti,ab,kw. | 44 |
| 6 | (insufflat* adj1 exsufflat*).ti,ab,kw. | 135 |
| 7 | MI-E.ti,ab,kw. | 76 |
| 8 | (direct* adj2 cough*).ti,ab,kw. | 60 |
| 9 | (cough* adj2 flow* adj5 (improv* or increas* or enhanc* or expan* or exten*)).ti,ab,kw. | 58 |
| 10 | (respiratory muscle* adj2 (aid* or support*)).ti,ab,kw. | 33 |
| 11 | (recruit* adj2 (lung volume or aveolar)).ti,ab,kw. | 116 |
| 12 | ((lung or alveolar) adj1 recruit* adj2 (manoeuv* or maneuv*)).ti,ab,kw. | 311 |
| 13 | 1 or 2 or 3 or 4 or 5 or 6 or 7 or 8 or 9 or 10 or 11 or 12 | 1018 |
| 14 | exp Animals/ not (exp Animals/ and Humans/) | 4706900 |
| 15 | (comment or editorial or letter or interview or news).pt. or (letter or editorial or comment).ti. or respiratory muscle training.ti,kw. | 2128982 |
| 16 | 13 not 14 not 15 | 849 |
| 17 | exp Pediatrics/ or (pediatr* or paediatr* or child* or newborn* or infant*).ti. | 1144485 |
| 18 | (exp Child/ or exp Infant/) not exp Adult/ | 1662619 |
| 19 | 16 not 17 not 18 | 715 |
| 20 | limit 19 to ed=20200224-20200615 | 19 |

EMBASE (OVID): Database(s): **Embase Classic+Embase**1947 to 2020 June 12
Search Strategy:

| **#** | **Searches** | **Results** |
| --- | --- | --- |
| 1 | (cough* adj2 assist*).ti,ab,kw. | 492 |
| 2 | (CoughAssist* or Pegaso* or Cofflator* or Cof-flator* or cough machine*).ti,ab,kw. | 116 |
| 3 | (cough* adj2 augment*).ti,ab,kw. | 115 |
| 4 | exp coughing/rh | 11 |
| 5 | (in-exsufflator* or in-exsufflation*).ti,ab,kw. | 79 |
| 6 | (insufflat* adj1 exsufflat*).ti,ab,kw. | 238 |
| 7 | MI-E.ti,ab,kw. | 140 |
| 8 | (direct* adj2 cough*).ti,ab,kw. | 81 |
| 9 | (cough* adj2 flow* adj5 (improv* or increas* or enhanc* or expan* or exten*)).ti,ab,kw. | 89 |
| 10 | (respiratory muscle* adj2 (aid* or support*)).ti,ab,kw. | 57 |
| 11 | (recruit* adj2 (lung volume or aveolar)).ti,ab,kw. | 194 |
| 12 | ((lung or alveolar) adj1 recruit* adj2 (manoeuv* or maneuv*)).ti,ab,kw. | 476 |
| 13 | 1 or 2 or 3 or 4 or 5 or 6 or 7 or 8 or 9 or 10 or 11 or 12 | 1651 |
| 14 | (exp animal experiment/ or exp animal model/ or nonhuman/ or exp vertebrate/) not (exp human/ or exp human experiment/) | 6693167 |
| 15 | 13 not 14 | 1486 |
| 16 | editorial/ or letter/ or (letter or editorial or comment).ti. or respiratory muscle training.ti,kw. | 1782014 |
| 17 | 15 not 16 | 1429 |
| 18 | exp pediatrics/ or (pediatr* or paediatr* or child* or newborn* or infant*).ti. | 1501891 |
| 19 | exp child/ not exp adult/ | 2214791 |
| 20 | 17 not 18 not 19 | 1196 |
| 21 | limit 20 to dd=20200224-20200615 | 26 |

CINAHL (EBSCO):

13 hits - Publicatiedatum: 20200201-20200631

| S17 | S15 NOT S16 |
| --- | --- |
| S16 | (MH "Animals+") NOT (MH "Human") |
| S15 | S13 not S14 |
| S14 | ( PT comment or editorial or letter or news ) OR TI ( comment or editorial or letter ) |
| S13 | S1 OR S2 OR S3 OR S4 OR S5 OR S6 OR S7 OR S8 OR S9 OR S10 OR S11 OR S12 |
| S12 | TI ( (lung or alveolar) N1 recruit* N2 (manoeuv* ormaneuv*) ) OR AB ( (lung or alveolar) N1 recruit* N2 (manoeuv* or maneuv*) ) |
| S11 | TI ( recruit* N2 (“lung volume” or alveolar) ) OR AB ( recruit* N2 (“lung volume” or alveolar) ) |
| S10 | TI ( respiratory muscle* N2 (aid* or support*) ) OR AB ( respiratory muscle* N2 (aid* or support*) ) |
| S9 | TI ( cough* N2 flow* N5 (improv* or increas* or enhanc* or expan* or exten*) ) OR AB ( cough* N2 flow* N5 (improv* or increas* or enhanc* or expan* or exten*) ) |
| S8 | TI direct* N2 cough* OR AB direct* N2 cough* |
| S7 | TI “MI-E” OR AB “MI-E” |
| S6 | TI insufflat* N1 exsufflat* OR AB insufflat* N1 exsufflat* |
| S5 | TI ( (in-exsufflator* or in-exsufflation*) OR AB ( (in-exsufflator* or in-exsufflation*) |
| S4 | (MH "Cough/RH") |
| S3 | TI cough* N2 augment* OR AB cough* N2 augment* |
| S2 | TI ( CoughAssist* or Pegaso* or Cofflator* or Cof-flator* or cough machine* ) OR AB ( CoughAssist* or Pegaso* or Cofflator* or Cof-flator* or cough machine* ) |
| S1 | TI cough* N2 assist* OR AB cough* N2 assist* |

Cochrane Library:

ID Search Hits

#1 (cough* near/2 assist*):ti,ab,kw 74

#2 (CoughAssist* or Pegaso* or Cofflator or Cof-flator* or (cough next machine*)):ti,ab,kw 26

#3 (cough* near/2 augment*):ti,ab,kw 29

#4 MeSH descriptor: [Cough] explode all trees and with qualifier(s): [rehabilitation - RH] 2

#5 (in-exsufflator* or in-exsufflation*):ti,ab,kw 20

#6 (insufflat* near/1 exsufflat*):ti,ab,kw 47

#7 (MI-E):ti,ab,kw 53

#8 (direct* near/2 cough*):ti,ab,kw 18

#9 ((cough* near/2 flow* near/5 (improv* or increas* or enhanc* or expan* or exten*))):ti,ab,kw 15

#10 (((respiratory next muscle*) near/2 (aid* or support*))):ti,ab,kw 4

#11 (recruit* near/2 (lung volume or alveolar)):ti,ab,kw 501

#12 (((lung or alveolar) near/1 recruit* near/2 (manoeuv* or maneuv*))):ti,ab,kw 182

#13 #1 or #2 or #3 or #4 or #5 or #6 or #7 or #8 or #9 or #10 or #11 or #12 678

#14 (respiratory muscle training):ti,ab,kw 1831

#15 #13 not #14 666

#16 (pediatr* or paediatr* or child* or newborn* or infant*):ti 100349

#17 #15 not #16 579

#18 MeSH descriptor: [Pediatrics] explode all trees 659

#19 #17 not #18 with Cochrane Library publication date Between Feb 2020 and Jun 2020, in Cochrane Reviews, Trials 26

WEB OF SCIENCE:

44 hits

Timespan: All years. Indexes: SCI-EXPANDED, SSCI, A&HCI, ESCI.

**TOPIC:**  ((((cough* NEAR/2 assist*) )  OR  ((CoughAssist* or Pegaso* or Cofflator* or Cof-flator* or cough machine*) )  OR  ((cough* NEAR/2 augment*) )  OR  (("in-exsufflator" or "in-exsufflators" or "in-exsufflation" or "in-exsufflations") )  OR  ((insufflat* NEAR/1 exsufflat*) )  OR  ("MI-E")  OR  ((direct* NEAR/2 cough*) )  OR  ((cough* NEAR/2 flow* NEAR/5 (improv* or increas* or enhanc* or expan* or exten*) ))  OR  ((("respiratory muscle" or "respiratory muscles")  NEAR/2  (aid* or support*) ))  OR  ((recruit* NEAR/2 ("lung volume" or alveolar) ))  OR  (((lung or alveolar)  near/1  recruit*  near/2  (manoeuv* or maneuv*) )))) *NOT* **TOPIC:**  (respiratory muscle training) *NOT* **TITLE:**  (pediatr* or paediatr* or child* or newborn* or infant*) *NOT* **TOPIC:**  ((animals NOT humans) ) *NOT* **DOCUMENT TYPES:**  (Bibliography OR Correction OR Correction, Addition OR Discussion OR Editorial Material OR Letter OR Meeting Abstract OR News Item OR Note)

**Refined by:** **PUBLICATION YEARS:** ( 2020 )

*Indexes=SCI-EXPANDED, SSCI, A&HCI, ESCI Timespan=All years*
